# Supplementary material for: Incidence of Myocardial Injury in COVID-19-Infected Patients: A Systematic Review and Meta-Analysis
Source: Diseases. 2020 Oct 27;8(4):40. doi: 10.3390/diseases8040040 (PMC7709098; doi:10.3390/diseases8040040)
Supplement: Supplementary file 1 [file diseases-08-00040-s001.pdf]

Supplementary data

Item 1. Search Strategy

Search term for OVID Medline

1. exp Coronavirus Infections/ or COVID.mp.
2. limit 1 to humans
3. exp Myocardial Ischemia/ or myocardial.mp.
4. 1 and 3

Search terms for EMBASE

No.	Query
#13	#11 AND 2020:py AND 'coronavirus infection'/dm
#12	#11 AND 2020:py
#11	#1 AND #10
#10	#2 OR #9
#9	#5 OR #8
#8	#4 AND #7
#7	#3 OR #6
#6	myocardial
#5	troponin
#4	injury
#3	cardiac
#2	clinical
#1	'covid' OR 'corona'

Database: PubMed

("COVID" OR "corona") AND ("clinical" OR "myocardial" OR "cardiac" OR "injury")

Search strategy

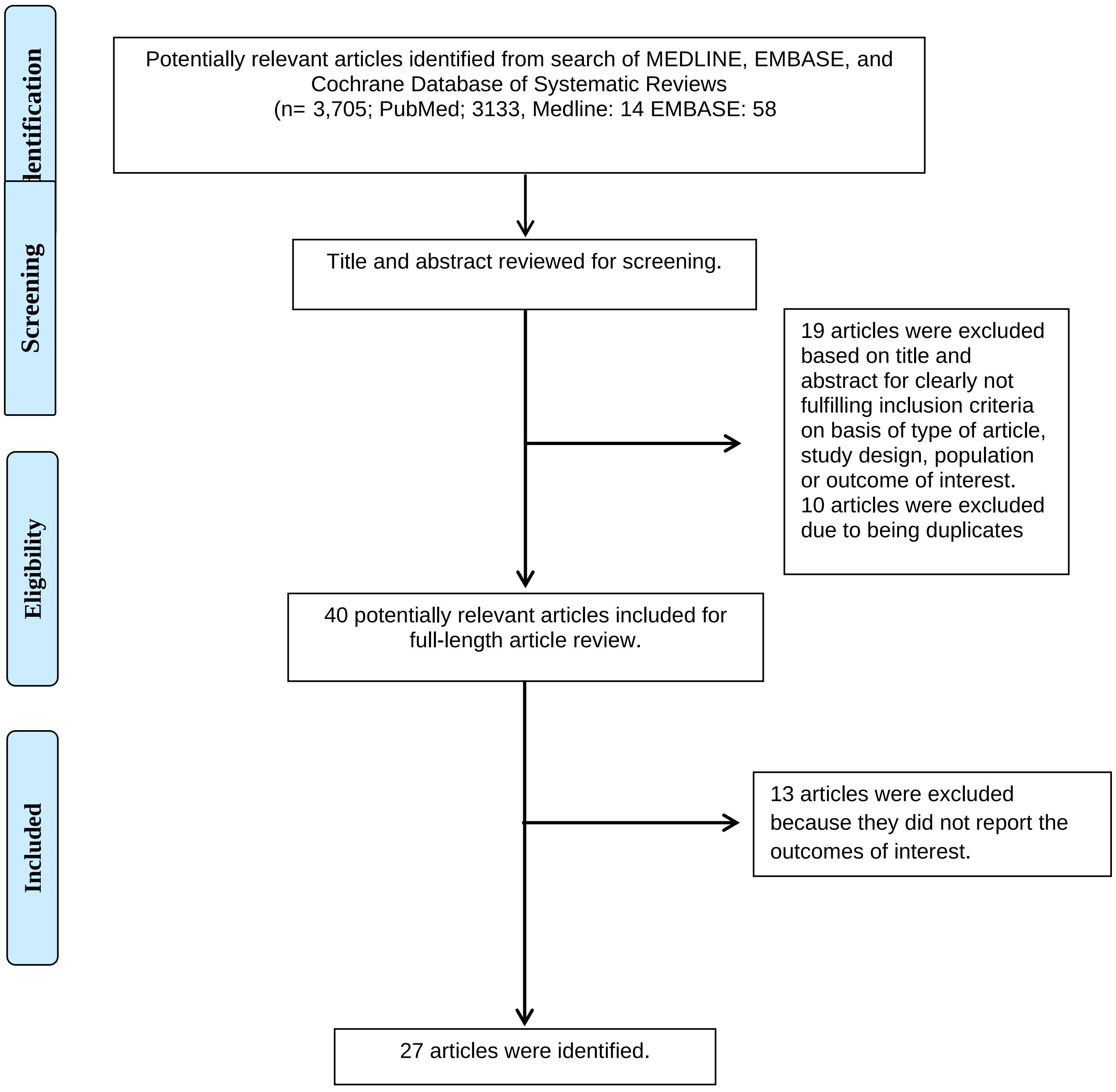

Supplement Table 1: Newcastle-Ottawa quality assessment scale of included studies in meta-analysis

Notes: The Newcastle-Ottawa scale uses a star system (0 to 9) to evaluate included studies on 3 domains: selection, comparability, and outcomes. Star (*)= item presents. Maximum 1 star (*) for selection and outcome components and 2 stars (**) for comparability components. Higher scores represent higher study quality.

Study	Selection				Comparability	Outcome			Total
	Representative of exposed cohort	Selection of the non-exposed cohort	Ascertainment of exposure	Endpoint not present at start	Comparability (Confounding)	Assessment of Outcome	Follow-up duration	Adequacy follow-up	
He[1]	*	*	*	*		*		*	6
Huang[2]	*	*	*	*	*	*	*	*	8
Wang[3]	*	*	*	*	*	*		*	7
Zhou[4]	*	*	*	*	*	*	*	*	8
Liu[5]	*	*	*	*	*	*			5
Chen[6]	*	*	*	*		*		*	6
Shi[7]	*	*	*	*		*		*	6
Deng[8]	*	*	*	*		*		*	6
Yang[9]	*	*	*	*		*			5
Wan[10]	*	*	*	*		*		*	6
Cao[11]	*	*	*	*		*			5
Guo[12]	*	*	*	*		*	*	*	7
Tao[13]	*	*	*	*		*			5
Tu[14]	*	*	*	*		*			5
Du[15]	*	*	*	*	*	*		*	6
Xu[16]	*	*	*	*	*	*		*	6
Wu[17]	*	*	*	*	*	*		*	6
Wei[18]	*	*	*	*		*			5
Ni[19]	*	*	*	*	*	*		*	6
Li[20]	*	*	*	*	*	*		*	6
Yu[21]	*	*	*	*	*	*			5
Feng[22]	*	*	*	*	*	*			6
Lombardi[23]	*	*	*	*	*	*	*	*	8
Javanian[24]	*	*	*	*		*			5
Saleh[25]	*	*	*	*		*			5

Chung[26]	*	*	*	*		*			5
Richardson[27]	*	*	*	*	*	*	*	*	8

Overall result

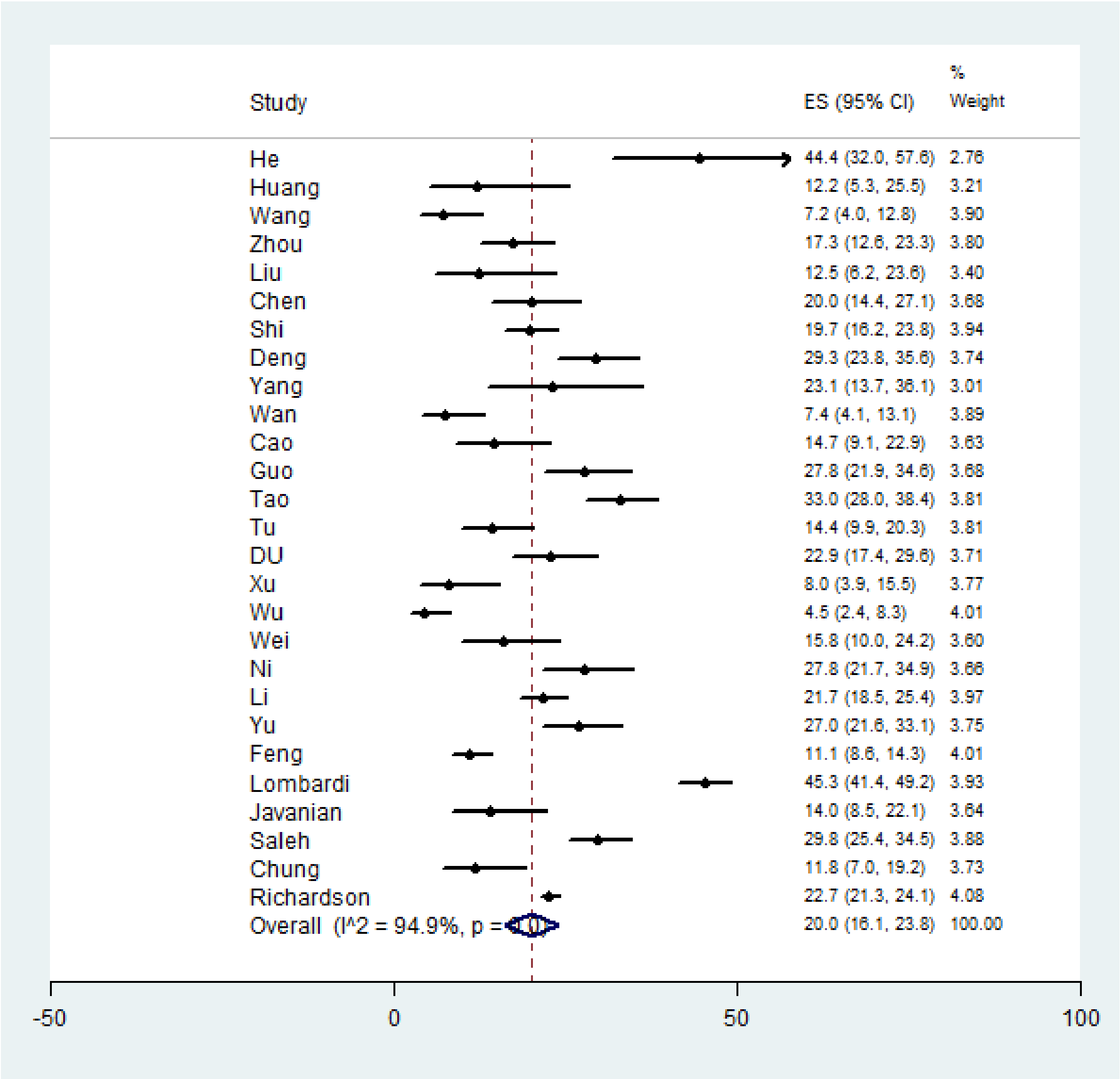

Meta-regression

By hypertension

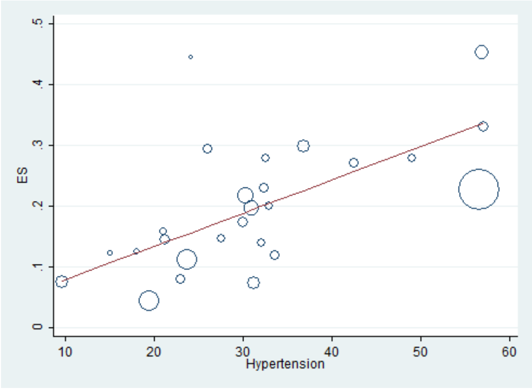

. . metareg _ES hypertension, wsse(_seES) graph

```
Meta-regression                                 Number of obs  =      26
REML estimate of between-study variance             tau2          = .004923
% residual variation due to heterogeneity          I-squared_res  =  91.35%
Proportion of between-study variance explained       Adj R-squared  =
50.87%
With Knapp-Hartung modification
------------------------------------------------------------------------------
         _ES |      Coef.   Std. Err.       t    P>|t|     [95% Conf. Interval]
-------------+----------------------------------------------------------------
hypertension |   .0054781   .0012524     4.37   0.000     .0028933    .0080629
       _cons |   .0240741   .0427272     0.56   0.578    -.0641105    .1122587
------------------------------------------------------------------------------

.
end of do-file
```

By age

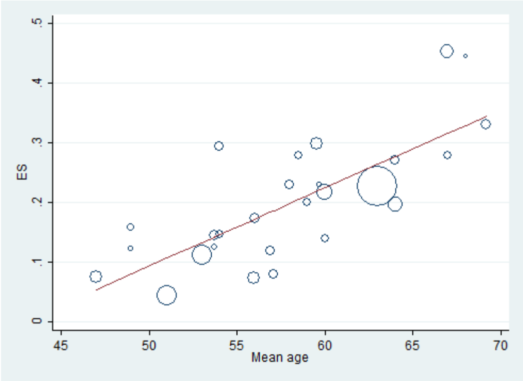

. . metareg _ES meanage, wsse(_seES) graph

```
Meta-regression                                 Number of obs  =      27
REML estimate of between-study variance             tau2          = .003988
% residual variation due to heterogeneity          I-squared_res  =  86.83%
Proportion of between-study variance explained       Adj R-squared  =  58.87%
With Knapp-Hartung modification
------------------------------------------------------------------------------
         _ES |      Coef.   Std. Err.       t    P>|t|     [95% Conf. Interval]
-------------+----------------------------------------------------------------
     meanage |   .0131335   .0023261     5.65   0.000     .0083428    .0179242
       _cons |  -.5634105   .1356138    -4.15   0.000    -.8427124   -.2841086
------------------------------------------------------------------------------

.
end of do-file

. do "C:\Users\narut\AppData\Local\Temp\STD00000000.tmp"
```

By sex (Male)

```
. . metareg _ES sexmale, wsse(_seES) graph

Meta-regression                          Number of obs  =      25
REML estimate of between-study variance          tau2          = .007419
% residual variation due to heterogeneity        I-squared_res  =  94.45%
Proportion of between-study variance explained      Adj R-squared  =  15.56%
With Knapp-Hartung modification
------------------------------------------------------------------------------
         _ES |      Coef.   Std. Err.      t    P>|t|     [95% Conf. Interval]
-------------+----------------------------------------------------------------
     sexmale |   .0050751   .0023335     2.17   0.040     .0002479    .0099024
       _cons |  -.0929421     .13121    -0.71   0.486    -.3643706    .1784865
------------------------------------------------------------------------------

.
end of do-file

. do "C:\Users\narut\AppData\Local\Temp\STD00000000.tmp"
```

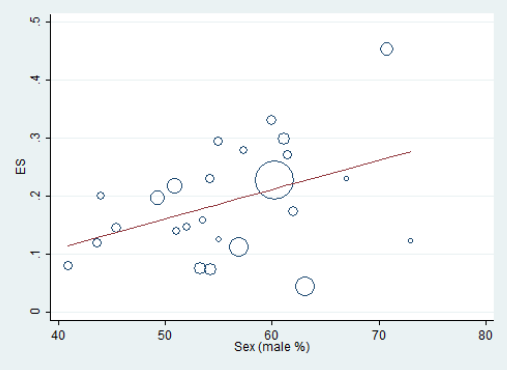

By diabetes

```
. . metareg _ES dm, wsse(_seES) graph

Meta-regression                          Number of obs  =      27
REML estimate of between-study variance          tau2          = .006805
% residual variation due to heterogeneity        I-squared_res  =  92.57%
Proportion of between-study variance explained      Adj R-squared  =  29.82%
With Knapp-Hartung modification
------------------------------------------------------------------------------
         _ES |      Coef.   Std. Err.      t    P>|t|     [95% Conf. Interval]
-------------+----------------------------------------------------------------
          dm |   .0062494   .0020571     3.04   0.006     .0020128    .0104861
       _cons |   .0905798   .0398137     2.28   0.032     .0085819    .1725777
------------------------------------------------------------------------------

.
end of do-file

. do "C:\Users\narut\AppData\Local\Temp\STD00000000.tmp"
```

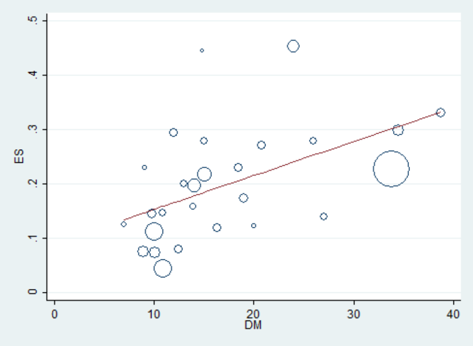

By coronary heart disease

```
. . metareg _ES chd, wsse(_seES) graph

Meta-regression                          Number of obs  =      27
REML estimate of between-study variance          tau2          = .007611
% residual variation due to heterogeneity        I-squared_res  =  92.60%
Proportion of between-study variance explained      Adj R-squared  =  21.50%
With Knapp-Hartung modification
------------------------------------------------------------------------------
         _ES |      Coef.   Std. Err.      t    P>|t|     [95% Conf. Interval]
-------------+----------------------------------------------------------------
         cvd |   .0066804   .0027627     2.42   0.023     .0009905    .0123703
       _cons |   .1231423   .0365366     3.37   0.002     .0478939    .1983908
------------------------------------------------------------------------------

.
end of do-file

. do "C:\Users\narut\AppData\Local\Temp\STD00000000.tmp"
```

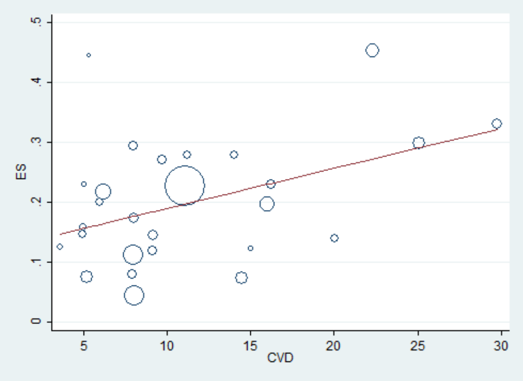

By CKD

```
. . metareg _ES ckd, wsse(_seES) graph

Meta-regression                              Number of obs  =      14
REML estimate of between-study variance            tau2          = .007579
% residual variation due to heterogeneity          I-squared_res  =  92.82%
Proportion of between-study variance explained       Adj R-squared  =  23.39%
With Knapp-Hartung modification
------------------------------------------------------------------------------
         _ES |      Coef.   Std. Err.      t    P>|t|     [95% Conf. Interval]
-------------+----------------------------------------------------------------
         ckd |    .010586   .0050606     2.09   0.058      -.00044    .0216121
       _cons |   .1693508   .0338024     5.01   0.000     .0957017    .2429998
------------------------------------------------------------------------------
```

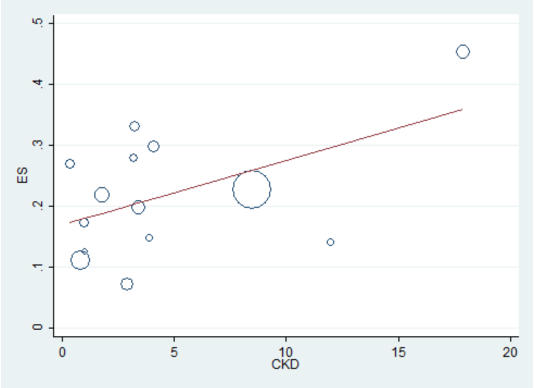

By region

```
. . metareg _ES nation, wsse(_seES) graph

Meta-regression                              Number of obs  =      27
REML estimate of between-study variance            tau2          = .009586
% residual variation due to heterogeneity          I-squared_res  =  94.42%
Proportion of between-study variance explained       Adj R-squared  =   1.13%
With Knapp-Hartung modification
------------------------------------------------------------------------------
         _ES |      Coef.   Std. Err.      t    P>|t|     [95% Conf. Interval]
-------------+----------------------------------------------------------------
      nation |   .0269995   .0255686     1.06   0.301    -.0256601    .0796591
       _cons |   .1644963   .0390281     4.21   0.000     .0841164    .2448762
------------------------------------------------------------------------------

.
end of do-file

. do "C:\Users\narut\AppData\Local\Temp\STD00000000.tmp"
```

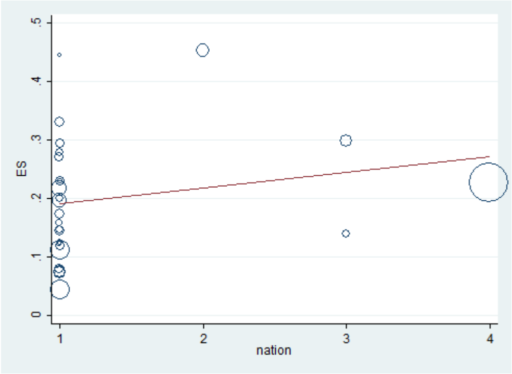

By race

```
. . metareg _ES race, wsse(_seES) graph

Meta-regression                              Number of obs  =      27
REML estimate of between-study variance            tau2          =  .00933
% residual variation due to heterogeneity          I-squared_res  =  93.71%
Proportion of between-study variance explained       Adj R-squared  =   3.78%
With Knapp-Hartung modification
------------------------------------------------------------------------------
         _ES |      Coef.   Std. Err.      t    P>|t|     [95% Conf. Interval]
-------------+----------------------------------------------------------------
        race |   .0436455    .034542     1.26   0.218    -.0274951    .1147861
       _cons |    .146169   .0468765     3.12   0.005      .049625    .2427129

------------------------------------------------------------------------------
```

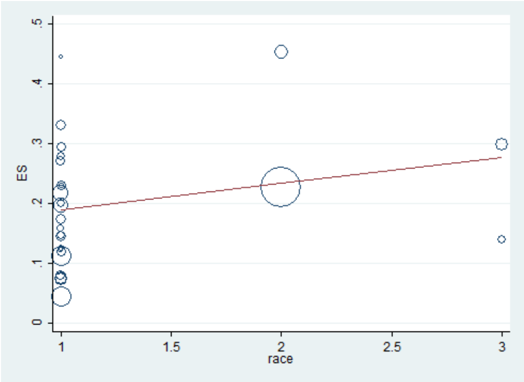

References

1. He XW, Lai JS, Cheng J, et al. [Impact of complicated myocardial injury on the clinical outcome of severe or critically ill COVID-19 patients]. *Zhonghua Xin Xue Guan Bing Za Zhi.* 2020;48(6):456-460.
2. Huang C, Wang Y, Li X, et al. Clinical features of patients infected with 2019 novel coronavirus in Wuhan, China. *Lancet.* 2020;395(10223):497-506.
3. Wang D, Hu B, Hu C, et al. Clinical Characteristics of 138 Hospitalized Patients With 2019 Novel Coronavirus-Infected Pneumonia in Wuhan, China. *JAMA.* 2020;323(11):1061-1069.
4. Zhou F, Yu T, Du R, et al. Clinical course and risk factors for mortality of adult inpatients with COVID-19 in Wuhan, China: a retrospective cohort study. *Lancet.* 2020;395(10229):1054-1062.
5. Liu K, Chen Y, Lin R, Han K. Clinical features of COVID-19 in elderly patients: A comparison with young and middle-aged patients. *J Infect.* 2020;80(6):e14-e18.
6. Chen C, Chen C, Yan JT, Zhou N, Zhao JP, Wang DW. [Analysis of myocardial injury in patients with COVID-19 and association between concomitant cardiovascular diseases and severity of COVID-19]. *Zhonghua Xin Xue Guan Bing Za Zhi.* 2020;48(7):567-571.
7. Shi S, Qin M, Shen B, et al. Association of Cardiac Injury With Mortality in Hospitalized Patients With COVID-19 in Wuhan, China. *JAMA Cardiol.* 2020;5(7):802-810.
8. Deng Y, Liu W, Liu K, et al. Clinical characteristics of fatal and recovered cases of coronavirus disease 2019 in Wuhan, China: a retrospective study. *Chin Med J (Engl).* 2020;133(11):1261-1267.
9. Yang X, Yu Y, Xu J, et al. Clinical course and outcomes of critically ill patients with SARS-CoV-2 pneumonia in Wuhan, China: a single-centered, retrospective, observational study. *Lancet Respir Med.* 2020;8(5):475-481.
10. Wan S, Xiang Y, Fang W, et al. Clinical features and treatment of COVID-19 patients in northeast Chongqing. *J Med Virol.* 2020;92(7):797-806.
11. Cao J, Hu X, Cheng W, Yu L, Tu WJ, Liu Q. Clinical features and short-term outcomes of 18 patients with corona virus disease 2019 in intensive care unit. *Intensive Care Med.* 2020;46(5):851-853.
12. Guo T, Fan Y, Chen M, et al. Cardiovascular Implications of Fatal Outcomes of Patients With Coronavirus Disease 2019 (COVID-19). *JAMA Cardiol.* 2020;5(7):811-818.
13. Li T, Lu L, Zhang W, et al. Clinical characteristics of 312 hospitalized older patients with COVID-19 in Wuhan, China. *Arch Gerontol Geriatr.* 2020;91:104185.
14. Tu WJ, Cao J, Yu L, Hu X, Liu Q. Clinicolaboratory study of 25 fatal cases of COVID-19 in Wuhan. *Intensive Care Med.* 2020;46(6):1117-1120.
15. Du RH, Liang LR, Yang CQ, et al. Predictors of mortality for patients with COVID-19 pneumonia caused by SARS-CoV-2: a prospective cohort study. *Eur Respir J.* 2020;55(5).
16. Xu X, Yu MQ, Shen Q, et al. Analysis of inflammatory parameters and disease severity for 88 hospitalized COVID-19 patients in Wuhan, China. *Int J Med Sci.* 2020;17(13):2052-2062.
17. Wu C, Chen X, Cai Y, et al. Risk Factors Associated With Acute Respiratory Distress Syndrome and Death in Patients With Coronavirus Disease 2019 Pneumonia in Wuhan, China. *JAMA Intern Med.* 2020;180(7):934-943.

18. Wei JF, Huang FY, Xiong TY, et al. Acute myocardial injury is common in patients with COVID-19 and impairs their prognosis. *Heart.* 2020;106(15):1154-1159.
19. Ni W, Yang X, Liu J, et al. Acute Myocardial Injury at Hospital Admission Is Associated With All-Cause Mortality in COVID-19. *J Am Coll Cardiol.* 2020;76(1):124-125.
20. Li X, Xu S, Yu M, et al. Risk factors for severity and mortality in adult COVID-19 inpatients in Wuhan. *J Allergy Clin Immunol.* 2020;146(1):110-118.
21. Yu Y, Xu D, Fu S, et al. Patients with COVID-19 in 19 ICUs in Wuhan, China: a cross-sectional study. *Crit Care.* 2020;24(1):219.
22. Feng Y, Ling Y, Bai T, et al. COVID-19 with Different Severities: A Multicenter Study of Clinical Features. *Am J Respir Crit Care Med.* 2020;201(11):1380-1388.
23. Lombardi CM, Carubelli V, Iorio A, et al. Association of Troponin Levels With Mortality in Italian Patients Hospitalized With Coronavirus Disease 2019: Results of a Multicenter Study. *JAMA Cardiol.* 2020.
24. Javanian M, Bayani M, Shokri M, et al. Clinical and laboratory findings from patients with COVID-19 pneumonia in Babol North of Iran: a retrospective cohort study. *Rom J Intern Med.* 2020;58(3):161-167.
25. Karbalai Saleh S, Oraii A, Soleimani A, et al. The association between cardiac injury and outcomes in hospitalized patients with COVID-19. *Intern Emerg Med.* 2020.
26. Chung SM, Ahn JH, Moon JS. Response: The Risk of Diabetes on Clinical Outcomes in Patients with Coronavirus Disease 2019: A Retrospective Cohort Study (Diabetes Metab J 2020;44:405-13). *Diabetes Metab J.* 2020;44(4):625-626.
27. Richardson S, Hirsch JS, Narasimhan M, et al. Presenting Characteristics, Comorbidities, and Outcomes Among 5700 Patients Hospitalized With COVID-19 in the New York City Area. *JAMA.* 2020;323(20):2052-2059.
